# Advancing access to CAR T-cell therapy: insights and real-world experience from a community oncology practice

**DOI:** 10.3389/fonc.2026.1712533

**Published:** 2026-02-16

**Authors:** Gary L. Simmons, Scott Cross, Elias C. Pittos

**Affiliations:** 1 Virginia Oncology Associates, Norfolk, VA, United States; 2 McKesson, Irving, TX, United States

**Keywords:** CAR T-cell therapy, community oncology, outpatient, patient access, program implementation, real-world outcomes

## Abstract

**Introduction:**

Chimeric antigen receptor (CAR) T-cell therapy has transformed the treatment of hematologic malignancies, offering durable remissions for patients with otherwise refractory disease. However, access to these therapies remains limited and primarily restricted to academic medical centers, contributing to significant geographic and demographic disparities in care delivery.

**Methods:**

We conducted a retrospective review of a community practice-based outpatient CAR T-cell therapy program, independent of academic or hospital affiliation, treating patients with relapsed hematologic malignancies. The implementation process, completed over six months, followed a structured series of steps to ensure safe and effective outpatient administration.

**Results:**

Between April 19, 2022, and December 18, 2024, 41 adult patients received outpatient CAR T-cell therapy. Products administered included liso-cel (n=19), ide-cel (n=12), axi-cel (n=8), and brex-cel (n=2). CRS occurred in 68% of patients, and ICANS in 22%. Hospital admission was required for 49% of patients, with 15% needing ICU care. Clinical remission rates at day 100 and 1 year were consistent with published data for each product. Patients receiving 4-1BB co-stimulatory domain CAR T-cells were observed to have numerically lower hospitalization rates (32%) compared to those receiving CD28-based products (100%). No unexpected safety concerns were observed under home-based monitoring.

**Conclusion:**

This analysis demonstrates that outpatient CAR T-cell therapy can be safely delivered in a community oncology practice setting, offering a viable solution to expand access and reduce geographic barriers. Despite clinical success, payer reluctance remains a significant barrier, highlighting the urgent need for policy reform to enable community-based delivery of advanced therapies.

## 1 Introduction

Chimeric antigen receptor (CAR) T-cell therapy has revolutionized the treatment landscape for hematologic malignancies, offering durable remissions for patients with otherwise refractory diseases. From 2017 to date, a total of seven CAR T-cell products have received regulatory approval from the Food and Drug Administration (FDA) for various hematologic malignancies: axicabtagene ciloleucel (axi-cel; Yescarta^®^), brexucabtagene autoleucel (brex-cel; Tecartus^®^), ciltacabtagene autoleucel (cilta-cel; Carvykti^®^), idecabtagene vicleucel (ide-cel; Abecma^®^) lisocabtagene maraleucel (liso-cel; Breyanzi^®^), odecabtagene autoleucel (obe-cel; Aucatzyl^®^), and tisagenlecleucel (tis-cel; Kymriah^®^). Over time, these therapies have evolved significantly in safety and administration.

Despite the promise of CAR T-cell therapy, only 25% of patients who are clinically eligible for CAR T-cells ultimately receive these therapies (1, 2). At present, access to treatment is limited and largely confined to academic medical centers (AMC) due to the complexity of administration and the need for specialized infrastructure. While nearly 85% of patients with cancer in the United States are managed in community treatment settings, fewer than 15% receive CAR T-cell therapy outside of academic centers, reflecting a significant misalignment between access to care and CAR T-cell availability (3, 4). This concentration of care within academic settings increases logistical challenges as patients are required to navigate and secure transportation, caregiver support, and lodging for extended periods of time (5). Limited access also exacerbates geographic and demographic disparities, as patients of African descent receive treatment at less than half the rate of White patients, and those living over two hours from a treatment center are 40% less likely to receive care (6).

As new immunotherapy-based cellular therapies continue to emerge, there is concern academic programs are less likely to have the bed capacity to admit and monitor patients for toxicities over several days. There are also financial concerns for inpatient reimbursement, especially through the Centers for Medicare and Medicaid Services (CMS), where coverage often falls short of meeting the full cost of therapy (7). As a result of these challenges, many institutions have transitioned treatment to outpatient delivery models, reflecting growing clinical experience and evolving safety protocols (8, 9). Encouragingly, this shift is supported by increasing confidence in the management and safety of CAR T-cell therapy, as reflected in the FDA’s removal of Risk Evaluation and Mitigation Strategies (REMS) and adoption of less restrictive monitoring parameters (10). As demand for CAR T-cell therapies grow, it is important that community-based oncology practices develop the infrastructure for safe and high-quality outpatient CAR T-cell therapy programs. With established patient relationships and local accessibility, community oncology practices are well-positioned to expand access and integrate CAR T-cell therapy into broader cancer care delivery.

This report highlights the experience of an independent outpatient community practice-based CAR T-cell therapy program, outlining the challenges and key steps for successful program implementation and describing patient treatment outcomes that demonstrate the feasibility and impact of bringing these advanced therapies closer to where patients live.

## 2 Methods

The outpatient CAR T-cell therapy program was developed leveraging the practice’s existing Foundation for the Accreditation of Cellular Therapy (FACT)-accredited autologous stem cell transplantation infrastructure and quality systems. Program implementation and initial product onboarding were completed over six months and followed a series of steps designed to ensure optimal safety, feasibility, and compliance with regulatory and accreditation standards, as described in **Table 1**.

**Table 1:** Crucial components for CAR T-cell outpatient program development and implementation.

Clearly defining the scope of service and catchment area	• Focus program development on FDA-approved CAR T-cell products. • Define patient eligibility criteria based on FDA-approved indications. • Identify geographic region served by the program.
Collaborating with key multidisciplinary stakeholders	• Discuss the outpatient program and workflow development with a working group of key stakeholders. • Stakeholders include physicians, pharmacists, nurses, advanced practitioners, pharmacy quality managers, billing specialists, and administration personnel.
Ensuring sufficient infrastructure and standard operating procedures	• Confirm dedicated space for cell infusion and monitoring. • Develop SOPs to ensure workflow consistency and regulatory compliance, and to guide protocols for candidate selection, apheresis, cell infusion, and treatment monitoring. • Standardize protocols for pre-infusion evaluation, including baseline laboratory assessments, infectious disease screening, and organ function testing. • Develop lymphodepleting chemotherapy standards in accordance with product-specific prescribing information.
Forging external partnerships	• If not available in-house, coordinate cell collection and storage activities through external facilities such as the American Red Cross. • Partner with regional hospital capable of inpatient and ICU-level care for CAR T patients
Providing training and education for the clinical care team and administrative staff	• Conduct education sessions with key stakeholders and staff, reviewing CAR T-cell referral workflows, reimbursement procedures, treatment protocols, and adverse event management. • Prioritize care coordination and cross-training in collaboration with local hospital partners, including but not limited to clinicians and staff from the emergency department, ICUs, neurology, and cardiology teams.
Providing training and education for the patient and their caregiver	• Implement a structured post-infusion home monitoring system to detect and manage CAR T-cell-related toxicities such as CRS and ICANS and assess for admission need. • Maintain continuous provider availability and establish coordination with the partnering local hospital to ensure timely intervention for clinical complications.
Becoming a qualified treatment site	• Establish site qualification processes with CAR T-cell therapy manufacturers to gain access to the product. • Assess for accreditation, taking into account quality infrastructure, payer network requirements, and resource availability.
Ensuring timely and adequate reimbursement for services rendered	• Assemble a dedicated team of revenue and managed care specialists to navigate reimbursement processes, including prior authorization, coding, billing, and single-case letter of agreements with commercial payers.

CAR, chimeric antigen receptor; CRS, cytokine release syndrome; FDA, Food and Drug Administration; ICANS, immune-effector cell-associated neurotoxicity syndrome; ICUs, intensive-care units; SOPs, standard operating procedures.

### 2.1 Program development and implementation

A working group of key multidisciplinary stakeholders, including physicians, pharmacists, nurses, advanced practitioners, pharmacist quality managers, billing specialists, and administration personnel convened to discuss the program and workflow development. Key early considerations focused on defining the scope of service to guide the program’s staffing and operational readiness, and evaluating infrastructure for clinical and operational components such as apheresis, cell infusion, patient monitoring, and billing services. Cell collection was coordinated through the American Red Cross, which managed apheresis and storage logistics in accordance with product-specific requirements.

A structured post-infusion home monitoring system was implemented to detect and manage CAR T-cell-related toxicities such as cytokine release syndrome (CRS) and immune-effector cell-associated neurotoxicity syndrome (ICANS) and assess for admission need. Continuous provider availability and established coordination with the partnering local hospital ensured timely intervention for clinical complications.

### 2.2 Patient selection and protocol

Patients eligible for CAR T-cell therapy were identified through referral or consultation. Given the retrospective and observational design of this report and the use of de-identified data only, the study was determined to be exempt after Institutional Review Board review. In order to be eligible for treatment, patients were required to have a caregiver, stay within 30 minutes of the infusion center, and participate in a remote-based home-monitoring program for a minimum of 30 days after infusion. Before initiating apheresis, prior authorization and single-case agreements were secured with commercial payers for outpatient CAR T-cell therapy and tocilizumab administration. After cell collection, patients were assessed for disease burden and the need for bridging therapy. Additionally, patients and their caregivers were required to attend a one-hour educational session on CAR T-cell therapy, reviewing the treatment journey, relevant toxicities as well as caregiver monitoring responsibilities. Treatment protocols outlined lymphodepleting chemotherapy regimens to be administered over three days in the outpatient office followed by CAR T-cell infusion, as prescribed by the respective product FDA label. Patients were sent home with a journal, and caregivers were instructed to check the patient’s temperature every eight hours. Caregivers were responsible for transporting patients to and from the clinic for appointments and ensured patients were adherent with their at-home medications. Caregivers also performed immune effector cell-associated encephalopathy (ICE) assessments every eight hours which included handwriting checks.

Patients were seen in the office daily for a minimum of seven days for laboratory assessments, vitals screenings, and physical exams. Grade 1–2 cytokine release syndrome (CRS) and immune-effector cell-associated neurotoxicity syndrome (ICANS) were managed with tocilizumab and steroids, respectively, with admission at the discretion of the cell therapy physician. After seven days, follow-up frequency was adjusted based on physician judgment.

For observed deaths, adjudication was performed by the treating oncologist based on direct clinical oversight of patient care and review of the complete patient medical record.

### 2.3 Clinical outcomes

The main safety outcomes analyzed included incidence of CRS and ICANS as well as hospital admission and intensive care unit (ICU) stays. CRS and ICANS were graded according to the American Society for Transplantation and Cellular Therapy (ASTCT) criteria. Key efficacy outcomes included complete response rates at 100 days and one year post-infusion.

### 2.4 Statistical analysis

Descriptive statistics were used to summarize baseline characteristics and clinical outcomes, including severity of adverse events and reasons for hospital admission. Response rates for key safety and efficacy outcomes, including CRS, ICANS, hospital admission, ICU stay, and treatment response at Day 100 and 1 year, were reported as proportions with 95% confidence intervals (CIs) calculated using the Clopper-Pearson exact method. Statistical analysis was conducted in Python (v3.11) using SciPy (v1.14).

## 3 Results

### 3.1 Patient characteristics and clinical outcomes

Between April 19, 2022, and December 18, 2024, forty-three patients were evaluated and identified as potential candidates for CAR T-cell therapy. Of these, a total of forty-one adult patients determined to be appropriate and eligible received outpatient treatment at the community practice site. Two patients were referred to academic treatment centers for follow-up due to payer coverage restrictions. **Table 2** summarizes patient baseline characteristics and **Table 3** outlines clinical outcomes. The median age of patients was 69 years (range: 38-88). Patients had received a median of two prior lines of therapy, and nine patients (22%) received bridging treatment. Prior to the infusion, all patients and caregivers were educated on CAR T-cell therapy and were instructed on the importance of remaining adherent to home monitoring protocols. A total of twenty patients required hospital admission (49%; 95% CI, 32.9–64.9%) with a median time to admission of 4 days, and a median length of stay of nine days. Six patients needed ICU care (15%; 95% CI, 5.6–29.2%). CRS occurred in twenty-eight of forty-one patients (68%; 95% CI, 51.9–81.9%), and ICANS occurred in nine of forty-one patients (22%; 95% CI, 10.6–37.6%). At one year after CAR T-cell infusion, seventeen patients (41%;95% CI, 26.3-57.9%) were in complete response, while eleven patients (27%; 95% CI, 14.2-42.9%) had passed away. **Table 4** outlines mortality attribution across the study population.

**Table 2:** Patient baseline characteristics and eligibility.

Patient characteristics	All Patients (N = 41)*	R/R DLBCL (N = 18)*	TFL to DLBCL (N = 7)*	R/R FL (N = 1)	R/R MCL (N = 1)	R/R B-cell ALL (N = 1)	R/R MM (N = 12)*	Primary refractory DLBCL (N = 1)*
Median age in years (range)	69 (38-88)	67 (38-87)	76 (63-78)	43 (43-43)	69 (69-69)	67 (67-67)	73 (53-88)	65 (62-67)
ECOG, n (%)								
0-1	20 (49)	7 (39)	2 (29)	1 (100)	1 (100)	0 (0)	9 (75)	0 (0)
2	11 (27)	7 (39)	2 (29)	0 (0)	0 (0)	1 (100)	1 (8)	0 (0)
3	10 (24)	4 (22)	3 (43)	0 (0)	0 (0)	0 (0)	2 (17)	1 (100)
Co-morbidities, n (%)								
HTN	21 (51)	9 (50)	6 (85)	0 (0)	0 (0)	1 (100)	4 (33)	1 (100)
Dyslipidemia	13 (32)	6 (33)	2 (28)	0 (0)	0 (0)	0 (0)	4 (33)	1 (100)
Osteoarthritis	7 (41)	1 (6)	3 (43)	0 (0)	1 (100)	0 (0)	2 (17)	0 (0)
DM	8 (20)	3 (17)	3 (43)	0 (0)	0 (0)	0 (0)	2 (17)	0 (0)
GERD	5 (12)	4 (22)	0 (0)	0 (0)	0 (0)	0 (0)	1 (8)	0 (0)
Depression	5 (12)	4 (22)	0 (0)	0 (0)	0 (0)	0 (0)	1 (8)	0 (0)
DVT	4 (10)	2 (11)	0 (0)	0 (0)	0 (0)	0 (0)	1 (8)	1 (100)
COPD	3 (7)	2 (11)	0 (0)	0 (0)	0 (0)	0 (0)	1 (8)	0 (0)
Anxiety	3 (7)	1 (6)	0 (0)	0 (0)	0 (0)	0 (0)	2 (17)	0 (0)
CKD	2 (5)	0 (0)	1 (14)	0 (0)	0 (0)	0 (0)	1 (8)	0 (0)
Bridging therapy type, n (%)								
Chemotherapy	8 (20)	5 (28)	2 (29)	0 (0)	0 (0)	0 (0)	1 (8)	0 (0)
BTK inhibitor	2 (5)	2 (11)	0 (0)	0 (0)	0 (0)	0 (0)	0 (0)	0 (0)
Proteosome inhibitor	1 (2)	0 (0)	0 (0)	0 (0)	0 (0)	0 (0)	1 (8)	0 (0)
None	32 (78)	12 (66)	5 (71)	1 (100)	1 (100)	1 (100)	11 (92)	1 (100)
Prior Lines of Therapy, n (%)								
1	8 (20)	7 (39)	0 (0)	1 (100)	0 (0)	0 (0)	0 (0)	0 (0)
2	21 (51)	9 (50)	5 (71)	0 (0)	1 (100)	0 (0)	5 (42)	1 (100)
3	8 (20)	1 (6)	2 (29)	0 (0)	0 (0)	1 (100)	4 (33)	0 (0)
4	3 (7)	1 (6)	0 (0)	0 (0)	0 (0)	0 (0)	2 (17)	0 (0)
6	1 (2)	0 (0)	0 (0)	0 (0)	0 (0)	0 (0)	1 (8)	0 (0)

*Percentages may not sum to 100 due to rounding.

ALL, acute lymphoblastic leukemia; BTK, Bruton’s kinase; CKD, chronic kidney disease; COPD, chronic obstructive pulmonary disorder; DM, diabetes mellitus; DLBCL, diffuse large B-cell lymphoma; DVT, deep vein thrombosis; ECOG, Eastern Cooperative Oncology Group; FL, follicular lymphoma; GERD, gastroesophageal reflux disease; GNR, gram-negative rod; HTN, hypertension; ICANS, immune effector cell-associated neurotoxicity syndrome; ICU, intensive care unit; MCL, mantle cell lymphoma; MM, multiple myeloma; R/R, relapsed or refractory; TFL, transformed follicular lymphoma.

**Table 3:** Patient clinical outcomes by CAR T-cell product.

Characteristic or clinical outcome	All patients (N = 41) n (%; 95% CI)*	Liso-cel (N = 19) n (%)*	Ide-cel (N = 12) n (%)*	Axi-cel (N = 8) n (%)*	Brex-cel (N = 2) n (%)*
Age, median (range)	69 (38–88)	66 (43–87)	73 (53–88)	76 (38–84)	68 (67–69)
Disease					
R/R DLBCL	18 (44)	13 (68)	0	5 (62)	0
TFL to DLBCL	7 (17)	4 (21)	0	3 (38)	0
R/R MCL	1 (2)	0	0	0	1 (50)
R/R B-cell ALL	1 (2)	0	0	0	1 (50)
R/R MM	12 (29)	0	12 (100)	0	0
R/R FL	1 (2)	1 (5)	0	0	0
Primary Refractory DLBCL	1 (2)	1 (5)	0	0	0
Median days from consult to infusion, (range)	68 (35-392)	88 (42-392)	62 (41-112)	63 (41-244)	46 (35-56)
Prophylactic steroids	8 (20)	0 (0)	0 (0)	8 (100)	0 (0)
Any grade CRS	28 (68; 51.9-81.9)	6 (32)	12 (100)	8 (100)	2 (100)
Grade 1^†^	15 (54)	3 (50)	5 (42)	6 (75)	1 (50)
Grade 2^†^	11 (39)	2 (33)	7 (58)	1 (13)	1 (50)
Grade 3+^†^	2 (7)	1 (17)	0 (0)	1 (13)	0 (0)
Any grade ICANS	9 (22; 10.6-37.6)	1 (5)	2 (17)	5 (63)	1 (50)
Grade 1^†^	6 (67)	1 (100)	2 (100)	3 (60)	0 (0)
Grade 2^†^	1 (11)	0 (0)	0 (0)	1 (20)	0 (0)
Grade 3+^†^	2 (22)	0 (0)	0 (0)	1 (20)	1 (100)
Hospital admissions within 30 days					
Number admitted within 72 hours^†^	20 (49; 32.9-64.9)	6 (32)	4 (33)	8 (100)	2 (100)
Admission day post-infusion, median (range)	8 (0)	2 (33)	4 (100)	2 (25)	0 (0)
Reason for admission (N)	4 (1–28)	9 (3–28)	2.5 (2–3)	4 (1–25)	4 (4–4)
Heart block	1	0	0	1	0
Caregiver issues	1	1	0	0	0
CRS	12	2	2	7	1
GNR sepsis	1	1	0	0	0
ICANS	3	0	2	0	1
Disease progression	2	2	0	0	0
Requiring ICU stay	6 (15; 5.6-29.2)	1 (5)	0 (0)	3 (38)	2 (100)
Clinical status at Day 100					
Complete response	30 (73; 57.1-85.8)	14 (74)	11 (92)	4 (50)	1 (50)
Stable disease	2 (5; 0.6-16.5)	1 (5)	0 (0)	1 (13)	0 (0)
Progressive disease	4 (10; 2.7-23.1)	2 (11)	0 (0)	2 (25)	0 (0)
Total deaths	5 (12; 4.1-26.2)	2 (11)	1 (8)	1 (13)	1 (50)
Clinical status at Year 1					
Complete Response	17 (41; 26.3-57.9)	9 (47)	3 (25)	4 (50)	1 (50)
Progressed or relapsed	3 (7; 1.5-19.9)	1 (5)	2 (17)	0 (0)	0 (0)
Total deaths	11 (27; 14.2-42.9)	4 (21)	2 (17)	4 (50)	1 (50)
Not yet evaluable	10 (24)	5 (26)	5 (42)	0 (0)	0 (0)

*Percentages may not sum to 100 due to rounding. ^†^Denominator is subset of patients experiencing that event.

ALL, acute lymphoblastic leukemia; CAR, chimeric antigen receptor; CRS, cytokine release syndrome; DLBCL, diffuse large B-cell lymphoma; FL, follicular lymphoma; GNR, gram-negative rod; ICANS, immune effector cell-associated neurotoxicity syndrome; ICU, intensive care unit; MCL, mantle cell lymphoma; MM, multiple myeloma; R/R, relapsed or refractory; TFL, transformed follicular lymphoma.

**Table 4:** Mortality attribution summary following CAR T-cell infusion.

Parameter	All patients (N = 41)	Liso-cel (N = 19)	Ide-cel (N = 12)	Axi-cel (N = 8)	Brex-cel (N = 2)
Total deaths, n (%; 95% CI)*					
Within 100 days	5 (12; 4.1-26.2)	2 (11)	1 (8)	1 (13)	1 (50)
Within 1 year	11 (27; 14.2-42.9)	4 (21)	2 (17)	4 (50)	1 (50)
Cause of death, n (%)					
Disease progression	9 (81)	4 (100)	2 (100)	3 (75)	0 (0)
GI bleed	1 (8)	0 (0)	0 (0)	1 (25)	0 (0)
Septic Shock	1 (8)	0 (0)	0 (0)	0 (0)	1 (100)
Deaths following ICU stay (%)*	1 (8)	0 (0)	0 (0)	0(0)	1 (50)
Median months to death after infusion (range)	3.8 (0.5-11.3)	4.5 (0.5-11.3)	6.5 (1.8-11.3)	4 (1.3-4.6)	0.9

*Percentages may not sum to 100 due to rounding.

GI, gastrointestinal; ICU, intensive care unit.

Nineteen patients were treated with liso-cel. The complete response rate was 74% at day 100. CRS occurred in six patients (32%) and was grade 1–2 in five patients. One patient with grade 4 CRS required intensive care for hypoxic respiratory failure. ICANS occurred in only one patient (5%) and was grade 1. Six patients were admitted to the hospital (32%): one for lack of caregiver support, one for sepsis, two for CRS management, and two for disease progression. At one year, nine of nineteen patients were in complete remission (47%) while four patients (21%) had passed away due to disease progression.

Twelve patients were treated with ide-cel. The complete response rate was 92% at day 100. All twelve patients had grade 1–2 CRS (100%) and two had grade 1 ICANS (17%). Four patients were admitted to the hospital (33%) at a median of 2.5 days: two for ICANS management, one for grade 2 CRS management, and one for grade 1 CRS and acute kidney injury. At one year, three patients were in remission (25%) and two patients (17%) passed away from disease progression.

Eight patients were treated with axi-cel and received prophylactic dexamethasone 10 mg on days 0, 1, and 2 after infusion. The complete response rate at day 100 was 50%. CRS occurred in all eight patients (100%) and was grade 1 in six patients, grade 2 in one, and grade 4 in one. ICANS occurred in five patients (63%) and was grade 1 in three, grade 2 in one and grade 4 in one. All patients required hospital admissions (100%) at a median 4 days after infusion and three patients required ICU care (38%). One patient had a bowel perforation on day 8 which required surgery, one patient developed third degree heart block on day 25 which required pacemaker placement and one patient developed cardiac shock on day 10. At one year, four of eight patients (50%) were in remission while four patients (50%) had passed away; three deaths were attributed to disease progression and one patient passed away due to gastrointestinal bleeding.

Two patients were treated with brex-cel. One patient was admitted to the hospital on day 5 for grade 1 CRS. The patient developed grade 4 ICANS (50%) requiring intensive care before being successfully treated with dexamethasone and anakinra. This patient was in complete remission at day 100 and at one year. The other patient was admitted to the hospital on day 4 for grade 2 CRS. Unfortunately, this patient worsened to grade 4 CRS and septic shock and expired on day 30. Both patients required hospital admission (100%).

Patient-reported outcomes were collected from patients or their caregivers following CAR T-cell therapy using standardized patient feedback and satisfaction surveys. All patients or caregivers completed the assessments, evaluating their overall treatment experience and satisfaction with care received. Across the cohort, responses were positive and no complaints or concerns were reported.

## 4 Discussion

The successful implementation of an outpatient CAR T-cell therapy program in a community practice setting requires the coordination of multiple stakeholders across clinical, administrative, and operational workstreams. A critical initial step is early engagement with a small working group of key participants, including physicians, pharmacists, nursing staff, and contracting/billing personnel to develop standardized protocols and define workflows. Early considerations such as staffing needs, expertise in cell therapy, infrastructure, contracting and billing, post-infusion monitoring, and adverse event management are foundational to a program’s safe and efficient launch. Logistical planning for apheresis is also critical, particularly in determining whether cell collection will be performed on site or outsourced to a third-party partner. Alternatively, some manufacturers offer “just-in-time” product distribution logistics that may provide a practical approach when long-term storage is not feasible. FACT accreditation is another important consideration for program development that carries implications for access and reimbursement. While FACT accreditation may not be necessary to safely deliver CAR T-cell therapy, third-party payers are increasingly incorporating accreditation status into provider network criteria and coverage determinations. As such, it is important for programs to evaluate the need for accreditation to ensure access and support reimbursement and payer approval.

The rates of CRS, ICANs, and clinical remission are consistent with clinical data published for each CAR T-cell product (11). Notably, no unexpected safety concerns were observed with patients under home-based monitoring. Electronic wearable devices were not utilized for patient monitoring. Patients were safely cared for with regular temperature monitoring, consistent caregiver support, and direct access to the on-call cell therapy team via a dedicated phone line. All patients requiring escalation of care were safely admitted directly to the hospital bypassing the emergency room within a few hours of symptom notification. There was one death in a patient with refractory B-cell ALL that developed grade 4 CRS and septic shock while admitted after CAR T-cell infusion.

Preliminary differences were observed in the need for hospitalization between CD28 and 4-1BB co-stimulatory domain CAR T-cells. Among ten patients receiving CD28 CAR T-cells (axi-cel and brex-cel), all ten (100%) required hospital admission. Among thirty-one patients receiving 4-1BB CAR T-cells (liso-cel and ide-cel), only ten (32%) required hospital admission. While these findings should be interpreted cautiously given the small sample size and potential for selection bias, they point to important implications for program design and the possibility that 4-1BB products may be more favorable for outpatient delivery, assuming comparable efficacy between product types. Further evaluation is warranted to confirm these observations.

An outpatient community practice program has the potential to significantly reduce overall treatment costs by minimizing hospital admissions. The median length of stay for inpatient CAR T-cell delivery can range from 12 to 16 days following infusion (12, 13). In our analysis, 49% of patients required hospitalization post-infusion, with a median inpatient stay of nine days. While our review does not directly measure financial outcomes, previous economic evaluations have highlighted the potential cost benefits of outpatient CAR T-cell delivery. An analysis of Medicare utilization and cost trends found that the average total cost for inpatient CAR T-cell delivery was $498,723, compared to $414,393 for outpatient administration, demonstrating a savings of $84,330 in favor of outpatient care (14). Similarly, previous work by Lyman et al. provides insight into the potential economic implications of outpatient CAR T-cell delivery in a non-academic treatment setting. Compared to inpatient administration, outpatient CAR T-cell treatment is associated with a $29,834 (56%) reduction in hospitalization costs (15). These findings demonstrate the potential for outpatient CAR T-cell administration to meaningfully reduce the economic burden of care.

In addition to cost savings, outpatient delivery of CAR T-cell therapy offers additional non-financial advantages that can benefit both patients and health-systems. With structured remote monitoring in the home setting, outpatient administration supports improved patient experience and quality of life by allowing for clinical monitoring within the comfort of a patient’s own home environment (16). This approach also mitigates the risk to nosocomial infections by minimizing inpatient time and exposure (17). Moreover, when compared to inpatient treatment, outpatient CAR T-cell therapy facilitates more efficient healthcare resource utilization by reducing bed occupancy and patient length of stay while maintaining similar safety and efficacy outcomes.

However, despite these advantages, outpatient delivery of CAR T-cells presents some important limitations. Successful implementation and delivery requires extensive logistical and operational infrastructure with clear protocols for rapid admission pathways and trained multidisciplinary teams, which can strain resources and place significant burden on smaller or less-resourced community-based treatment sites. This model of care also shifts significant responsibility to caregivers, who must frequently monitor vital signs, recognize neurologic and systemic side effects of therapy, coordinate transportation, and remain near the treatment center, often for an extended period of time. Additionally, patient comprehension and adherence to monitoring instructions is critical. Frequent clinic visits can be financially burdensome and poor health literacy or lack of adequate caregiver support can restrict eligibility and increase risk.

One of the most significant obstacles in community outpatient delivery of CAR T-cells is the reluctance of commercial payers to authorize treatment outside of academic or inpatient facilities. Raising awareness among payers about the ability of capable and well-prepared community practices to safely and effectively deliver CAR T-cell therapy is essential. Despite being a fully equipped local program with active FACT accreditation, challenges with commercial payer authorization were encountered. Two patients were denied coverage solely because CAR T-cell administration was planned at a community-based practice site. As a result, both patients were required to travel and stay near an academic center more than 95 miles away to receive treatment. This is both unacceptable and unsustainable, especially in the context of a therapy that is intended to be curative, limiting access to care while increasing the financial burden for patients and their caregivers. There needs to be a fundamental change at how community practices are viewed by commercial payers, with a recognition that the community can open access to CAR T-cell therapy for patients by treating them locally and at a potential cost-savings to the patient, payer, and overall healthcare system.

To our knowledge, this is the first report describing the successful implementation and real-world outcomes of an independent community oncology practice-based outpatient CAR T-cell therapy program that is not affiliated with any academic center or hospital system. This analysis demonstrates that with appropriate infrastructure, multidisciplinary teams, and safety protocols, CAR T-cell therapy can be delivered safely and efficiently in this setting. It is our hope that that this experience can serve as a model for other community-based practices seeking to develop their own programs. As demand for CAR T-cell therapy grows, it is imperative that capable and well-prepared oncology community practices develop these programs locally to overcome treatment barriers and achieve scalable and more accessible care for patients.

## Data Availability

The original contributions presented in the study are included in the article/supplementary material. Further inquiries can be directed to the corresponding author.
